# *PALB2* germline mutations in a large cohort of Middle Eastern breast-ovarian cancer patients

**DOI:** 10.1038/s41598-023-34693-9

**Published:** 2023-05-11

**Authors:** Abdul K. Siraj, Rong Bu, Sandeep Kumar Parvathareddy, Kaleem Iqbal, Saud Azam, Zeeshan Qadri, Maha Al-Rasheed, Wael Haqawi, Mark Diaz, Ingrid G. Victoria, Ismail A. Al-Badawi, Asma Tulbah, Fouad Al-Dayel, Dahish Ajarim, Khawla S. Al-Kuraya

**Affiliations:** 1 Human Cancer Genomic Research, Research Center, King Faisal Specialist Hospital and Research Center, MBC#98-16, P.O. Box 3354, Riyadh, 11211 Saudi Arabia; 2 Department of Obstetrics-Gynecology, King Faisal Specialist Hospital and Research Center, Riyadh, 11211 Saudi Arabia; 3 Department of Pathology, King Faisal Specialist Hospital and Research Centre, Riyadh, 11211 Saudi Arabia; 4 Oncology Center, King Faisal Specialist Hospital and Research Centre, Riyadh, 11211 Saudi Arabia

**Keywords:** Cancer genetics, Breast cancer, Cancer genomics

## Abstract

The *PALB2* gene is a breast cancer (BC) and ovarian cancer (OC) predisposition gene involved in the homologous recombination repair pathway. However, the prevalence and clinicopathological association of *PALB2* pathogenic/likely pathogenic (PV/LPV) variants in Middle East is still not fully explored. Total 918 BC/OC patients from Saudi Arabia were selected for *PALB2* mutations screening using capture sequencing technology. Five heterozygous PVs or LPVs were identified in six cases, accounting for 0.65% (6/918) of entire cohort. Two cases (33.3%) harbored PVs and four cases (66.7%) carried LPVs. Four PVs/LPVs (80%) were frameshift along with one novel splicing LPV (c.2835-2_2835-1delinsTT). One recurrent LPV (c.3425delT: p.L1142fs) was identified in two cases. All six affected carriers have breast cancer diagnosis with median age of 39.5 years (range 34–49 years). Only two cases (33%) have documented family history of cancer. Breast cancer phenotype was invasive ductal unilateral cancer in all cases with 66.7% of hormone receptor positive and 16% of triple negative tumors. Germline PVs/LPVs in the *PALB2* gene were observed in low frequency of 0.65% in Saudi BC and/or OC. Our study confirms one recurrent LPV and one novel LPV in Saudi breast cancer patients.

## Introduction

Breast cancer is the leading cause of cancer mortality in women worldwide^1^. In Saudi Arabia, breast cancer is commonest cancer affecting Saudi females^2^. 5–10% of breast cancer and 10–15% ovarian cancer are thought to be hereditary^3–6^.

*BRCA1/2* germline pathogenic variants (PVs) and likely pathogenic variants (LPVs) are known to be the main susceptibility genes in hereditary breast and ovarian cancer (HBOC)^5,7,8^. The Partner and Localizer of *BRCA2* (*PALB2)* gene plays a critical role in homologous recombination repair of double standard DNA breaks for checkpoint control function through its ability to recruit *BRCA2* and *RAD51* to DNA breaks^9–14^.

Recently, *PALB2* has been identified as one of the common predisposing genes for breast cancer after *BRCA1/2* with penetrance estimated at 33–70% depending on age at diagnosis and family history^15–18^. Germline PVs/LPVs in *PALB2* have also been identified in ovarian cancer and pancreatic cancer patients^19,20^. The prevalence of germline *PALB2* PV/LPV in hereditary HBOC patients ranges between 0.8 and 1.5%^21–23^.

However, data about the prevalence of germline *PALB2* variants in breast and ovarian cancers from Middle Eastern ethnicity is still scarce. Therefore, we have conducted this study in a cohort of 918 breast and ovarian cancer patients from Middle Eastern ethnicity to investigate the prevalence of germline *PALB2* PVs and LPVs in breast cancer and ovarian cancer patients, and their molecular and clinicopathological characteristics in this population.

In addition, identifying the presence of recurrent and/or none *PALB2* PVs/LPVs will help in understanding the contribution to breast cancer and ovarian cancer risk and tailor the best preventive and treatment option of *PALB2* carriers from Middle Eastern ethnicity.

## Materials and methods

### Study population

The cases comprised 918 patients, 791 with breast cancer and 127 with epithelial ovarian cancer. All patients were of Saudi origin, diagnosed and treated at King Faisal Specialist Hospital and Research Centre (KFSH&RC) from 1989 to 2016. Relaxed criteria were used to select high-risk patients. For breast cancer, the criteria used were age ≤ 50 years, positive family history of cancer, triple negative breast cancers or bilateral tumors. Similarly, age ≤ 50 years, along with positive family history of cancer were considered high risk for ovarian cancer^24^. In our cohort of 918 breast and ovarian cancer patients, 271 patients had a positive family history of cancer in first and/or second-degree relatives. Among the patients with positive family history, 84.5% (229/271) had a relative with one of the well-known hereditary cancers (breast, ovarian, uterine, gastrointestinal, pancreatic, brain or soft tissue sarcomas).

Detailed clinico-pathological data, including follow-up data, were noted from case records and summarized in Table 1 (breast cancer) and Table 2 (epithelial ovarian cancer). The Institutional Review Board of King Faisal Specialist Hospital and Research Centre approved this study and the Research Advisory Council (RAC) of King Faisal Specialist Hospital and Research Centre provided waiver of informed consent under project RAC # 2140 008, since only retrospective patient data were analyzed. It is confirmed that all methods were performed in accordance with the relevant guidelines and regulations.

**Table 1:** Summary of clinico-pathological variables in breast cancer patients (n = 791).

Clinico-pathological variable	n (%)
**Age at diagnosis, years**	
Mean ± SD	40.9 ± 9.8
Median (range)	39 (13–84)
≤ 30	77 (9.8)
31–40	387 (48.9)
41–50	220 (27.8)
51–60	68 (8.6)
> 60	39 (4.9)
Family history of cancer	
No	529 (66.9)
Yes	262 (33.1)
Bilateral breast cancer	
Yes	15 (1.9)
No	776 (98.1)
Histologic grade	
Well differentiated	50 (6.3)
Moderately differentiated	342 (43.2)
Poorly differentiated	360 (45.5)
Unknown	40 (5.0)
Tumor size	
T1	208 (26.3)
T2	329 (41.6)
T3	131 (16.6)
T4	101 (12.8)
Unknown	22 (2.7)
Lymph node status	
Negative	294 (37.2)
Positive	475 (60.1)
Unknown	22 (2.7)
Distant metastasis	
Absent	697 (88.1)
Present	72 (9.2)
Unknown	22 (2.7)
Stage	
I	111 (14.0)
II	317 (40.1)
III	269 (34.0)
IV	72 (9.2)
Unknown	22 (2.7)
ER	
Positive	439 (55.5)
Negative	352 (44.5)
PR	
Positive	401 (50.7)
Negative	390 (49.3)
Her-2 neu	
Positive	254 (32.1)
Negative	537 (67.9)
Triple negative breast cancer	
Yes	199 (25.2)
No	592 (74.8)

**Table 2:** Summary of clinico-pathological variables in epithelial ovarian cancer patients (n = 127).

	n (%)
Age	
Mean ± SD	39.7 ± 9.8
Median (range)	41.3 (17–66)
≤ 30	30 (23.6)
31–40	29 (22.8)
41—50	59 (46.5)
51–60	8 (6.3)
> 60	1 (0.8)
Family history of cancer	
No	118 (92.9)
Yes	9 (7.1)
Histopathology	
High grade serous	56 (44.1)
Low grade serous	27 (21.3)
Mucinous	20 (15.7)
Endometrioid	16 (12.6)
Clear cell	3 (2.4)
Undifferentiated	5 (3.9)
Histological grade	
Grade 1	33 (26.0)
Grade 2	53 (41.7)
Grade 3	40 (31.5)
Unknown	1 (0.8)
pT	
T1	29 (22.8)
T2	13 (10.2)
T3	85 (67.0)
pN	
N0	120 (94.5)
N1	7 (5.5)
pM	
M0	105 (82.7)
M1	22 (17.3)
Stage	
I	29 (22.8)
II	9 (7.1)
III	67 (52.8)
IV	22 (17.3)

### DNA extraction

DNA samples were extracted from formalin-fixed and paraffin-embedded (FFPE) normal tissues of breast cancer or ovarian cancer patients utilizing Gentra DNA Isolation Kit (Gentra, Minneapolis, MN, USA) according to the manufacturer’s protocols as described in the previous study^25^. Two pathologists examined the histopathology slides to ensure that Normal tissues were obtained from different FFPE blocks such as uninvolved lymph nodes or non-cancerous breast tissue away from the tumor in order to minimize somatic contamination.

### Capture sequencing

Targeted capture sequencing was performed on 918 samples using Illumina platform with the custom designed panel and all the quality metrics were applied as described previously^26^. ACMG/AMP 2015 guideline and ClinVar were utilized for interpretation of pathogenicity of variants.

### Haplotype analysis

Haplotype analysis was performed using PHASE version 2.1.1 algorithm^27,28^. Number of variant positions, variant nucleotide positions and genotypes for each sample at those positions were supplied as an input in the algorithm. Following default parameter were set: number of iterations = 100, thinning interval = 1, burn-in = 100.

## Results

### Molecular results

**Figure 1:**
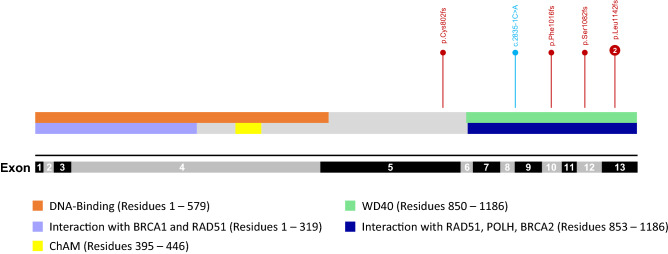
Schematic representation of the PALB2 protein with variant positions and domains. Pins represent mutation position. Number represents the number of cases with the mutation (unmarked pin represents 1 case). Red pins represent frameshift mutations; blue pin represents splice-site mutation.

In the entire cohort, five different types of PVs or LPVs were identified in six cases, accounting for 0.65% (6/918) of all analyzed cases. Among these five variants, four (80%) were frameshift variants while one (20%) was splicing variant. All four frameshift variants were reported previously as PV or LPV in breast cancer cases while the splicing LPV was novel (Table 3). The PV/LPV distribution in the *PALB2* gene was presented in the Fig. 1.

**Table 3:** Pathogenic/ Likely pathogenic *PALB2 (NM_024675.4)* variants in breast cancer.

	Chr	Start	End	Ref	Alt	HGVS	ClinVar	Mutation type
1	chr16	23,614,916	23,614,916	A	–	c.3425delT:p.L1142fs	Likely pathogenic	Frameshift deletion
2	chr16	23,641,068	23,641,069	CA	–	c.2406_2407del:p.C802fs	Pathogenic	Frameshift deletion
3	chr16	23,619,290	23,619,291	CT	–	c.3244_3245del:p.S1082fs	Pathogenic	Frameshift deletion
4	chr16	23,634,452	23,634,453	CT	AA	c.2835-2_2835-1delinsTT	NA	Splicing variant
5	chr16	23,632,748	23,632,748	A	–	c.3048delT:p.F1016fs	Likely pathogenic	Frameshift deletion

In the six mutant cases, all cases were breast cancer. None of ovarian cancer cases was found to carry any PVs or LPVs in *PALB2*. Only one recurrent LPV, NM_024675.4:c.3425del:p.Leu1142fs was identified. This variant was present in two unrelated cases. Interestingly, this homozygous *PALB2* p.Leu1142fs variant was also previously reported in two Fanconi Anemia families in our population with strong family history of multiple cancer types including Wilms tumor, neruoblastoma and acute myeloid leukemia^29^. Therefore, haplotype analysis was performed for these two cases utilizing Capture Sequencing data. However, the founder mutation could not be confirmed due to the limited available SNPs information extracted from capture sequencing data. In addition, it was first time that the splicing variant c.2835-2_2835-1delinsTT was reported as LPV in cancer patients. However, other PALB2 PVs or LPVs detected in our cohort were previously reported in breast cancer or ovarian cancer patients in the literature^30^ (Table 3).

### Clinico-pathological characteristics of breast cancer patients with PALB2 PV/LPVs

Breast cancer phenotype was invasive ductal (100%) with 66.7% of hormone-receptor positive and 16.7% of triple negative tumors. All six patients had either grade 2 (66.7%) or grade III (33.3%) tumors, with majority being stage II (66.7%). Family history was positive in 33.3% (2/6) of germline *PALB2* PV/LPV carriers. One of the patients had a sister with breast cancer, whereas another patient’s mother (liver cancer) and sister (breast cancer) had history of cancer. None of the patients had a personal history of other cancers. One of the patient died due to disease progression (metastasis to lung, liver, bone and brain) (Table 4).

**Table 4:** Clinico-pathological details of *PALB2* mutated cases in breast cancer (n = 6).

S no	Age	pT	pN	pM	Stage	Grade	Histology	ER	PR	Her-2	TNBC	BRCA 1/2 mutation	Other tumors in patient	Family history	Status
1	40	T4	N2	M0	III	2	IDC	Positive	Positive	Positive	No	Negative	No	Negative	Alive
2	38	T2	N2	M1	IV	3	IDC	Negative	Negative	Positive	No	Negative	No	Negative	Dead
3	39	T1	N1	M0	II	2	IDC	Positive	Positive	Negative	No	Negative	No	Positive—sister had breast cancer	Alive
4	41	T2	N0	M0	II	2	IDC	Negative	Negative	Negative	Yes	Negative	No	Positive—mother had liver cancer and sister had breast cancer	Alive
5	34	T3	N0	M0	II	3	IDC	Positive	Positive	Negative	No	Negative	No	Negative	Alive
6	49	T2	N0	M0	II	2	IDC	Positive	Positive	Positive	No	Negative	No	Negative	Alive

## Discussion

We identified six cases harboring *PALB2* PVs or LPVs among 918 breast and ovarian cancer patients. The previously reported frequency rate range of *PALB2* PV/LPV is from 0.1 to 1.5% depending on the population, cohort size and method of testing^22,31–34^. The frequency of *PALB2* PV/LPV in this cohort is 0.65%, and therefore lies within the lowest frequencies reported worldwide.

In current study, all the *PALB2* PVs/LPVs were identified in breast cancer cases and no *PALB2* PVs or LPVs were found in ovarian cases. However, this should be interpreted with caution since the ovarian cancer cohort is small in comparing to breast cancer cohort included in this study.

The one recurrent frameshift LPV p.Leu1142fs was found in two unrelated young patients with breast cancer. Both patients have a family history of breast cancer. This recurrent *PALB2* LPV identified in our cohort is particularly interesting since it was previously reported in two Fanconi anemia families with strong family history of multiple cancer types from Saudi Arabia 2016^29,35^. We also found one submission of this alteration in the ClinVar database from different laboratories reporting germline testing of hereditary breast cancer (undisclosed ancestry)^30^. However, haplotype analysis based on the limited available SNPs data of two cases harboring this LPV did not confirm founder effect. Therefore, further analysis to confirm the founder effect of this LVP is needed. In addition, the patient carrying the novel LPV c.2835-2_2835-1delinsTT did not have family history of breast cancer.

Molecular and clinicopathological characteristics of breast cancer cases carrying *PALB2* PVs/LPVs showed that all breast cancer tumors were invasive ductal carcinoma. Majority of the tumors were histologically intermediate to high grade, positive for HER2, ER and PR expression. Young onset was apparent in all the *PALB2* PV or LPV carries with all of them under the age of 50 years of age, including 4 under the age of 40.

This study has limitation; first, it is retrospective single center study where selection bias can’t be excluded. Second, the ovarian cancer cohort is relatively small to draw a conclusion regarding the absence of *PALB2* PV/LPV in ovarian cancer from this ethnicity. Third, germline filtering by peripheral blood sample could not be performed, due to unavailability of such samples.

In summary, this study was able to identify the prevalence of *PALB2* PV/LPV and describe the molecular and clinical characteristics of *PALB2* PV/LPV carriers in Saudi Arabia. Frequency of *PALB2* PV/LPV in breast cancer seems to be lower than in other population^31–34^. *PALB2* c.3425delT:p.Leu1142fs is a recurrent LPV that seems to be responsible for one third of *PALB2* mutant cases. Recent studies demonstrated that PARP inhibitor is effective in treating breast cancer patients carrying PALB2 PV or LPV, opening a new avenue of target treatment that might benefit breast cancer patients^36,37^. Genetic testing for relevant genes such as *PALB2* must be included in molecular and genetic evaluation of breast cancer patient from this population, which could contribute, to a better understanding of breast cancer risk and implementing preventive and therapeutic strategies for breast cancer patients from Middle Eastern ethnicity.

## Data Availability

All the data generated or analyzed during this study are included in this published article.
